# Supplementary material for: Targeted gene therapy and cell reprogramming in Fanconi anemia
Source: EMBO Mol Med. 2014 May 23;6(6):835–48. doi: 10.15252/emmm.201303374 (PMC4203359; doi:10.15252/emmm.201303374)
Supplement: Supplementary file 1 — Supplementary Figure S1 [file emmm0006-0835-sd1.pdf]

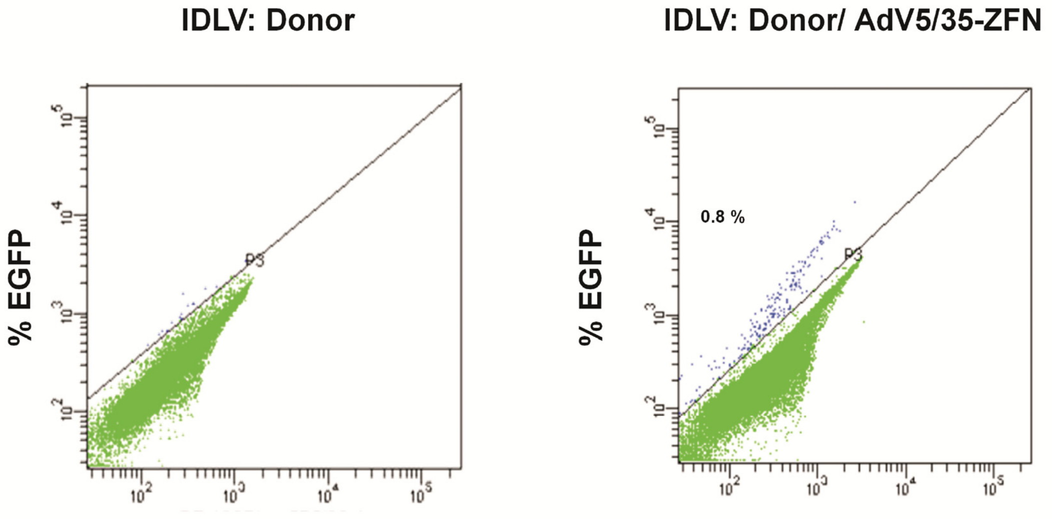

Figure S1: Representative flow cytometry dot plots of FA-A fibroblasts 14 days after transduction of FA fibroblasts with the donor IDLV ± the AdV5/35-ZFNs targeting the *AAVS1* locus. Left panel FA fibroblasts transduced with the donor IDLV alone and right panel donor IDLV + AdV5/35-ZFNs targeting the *AAVS1* locus.
