# Supplementary material for: Targeted gene therapy and cell reprogramming in Fanconi anemia
Source: EMBO Mol Med. 2014 May 23;6(6):835–48. doi: 10.15252/emmm.201303374 (PMC4203359; doi:10.15252/emmm.201303374)
Supplement: Supplementary file 2 — Supplementary Figure S2 [file emmm0006-0835-sd2.pdf]

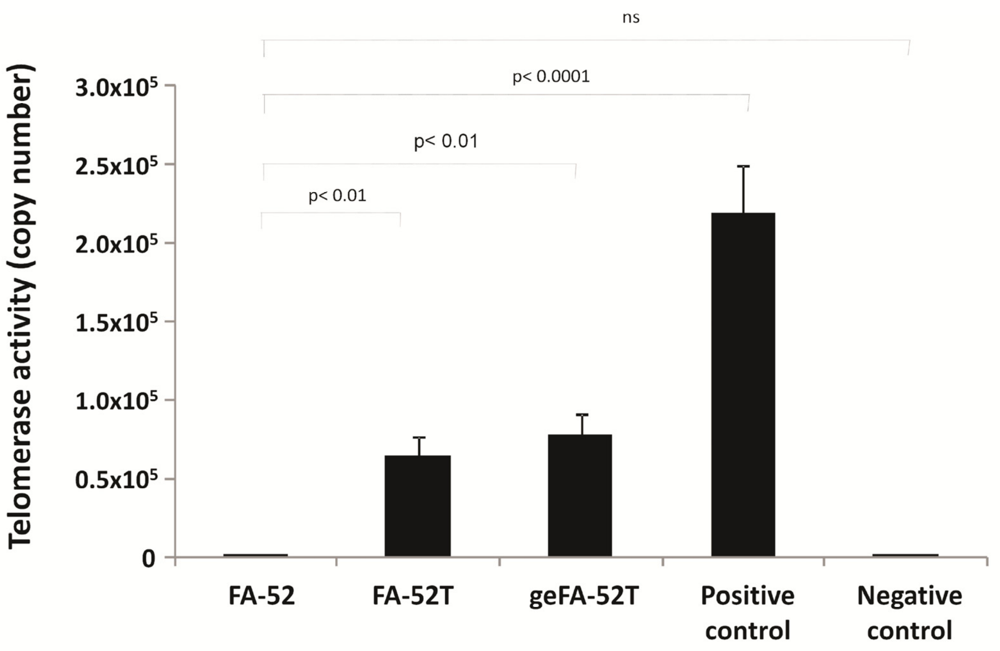

Figure S2: Telomerase activity in TERT-transduced FA fibroblasts: Analysis of the telomerase activity in FA fibroblasts prior to and after transduction with hTERT-LVs (passage 12 after transduction). Data corresponding to hTERT-transduced fibroblasts subjected to gene editing is also shown. A positive telomerase control and a minus telomerase control were included.

Data information: The figure represents mean and s.e. corresponding to triplicate analyses. $p < 0.0001$ by one-way ANOVA. Post Hoc Bonferroni's multiple comparison is shown. ns: not significant.
