# Supplementary material for: Targeted gene therapy and cell reprogramming in Fanconi anemia
Source: EMBO Mol Med. 2014 May 23;6(6):835–48. doi: 10.15252/emmm.201303374 (PMC4203359; doi:10.15252/emmm.201303374)
Supplement: Supplementary file 3 — Supplementary Figure S3 [file emmm0006-0835-sd3.pdf]

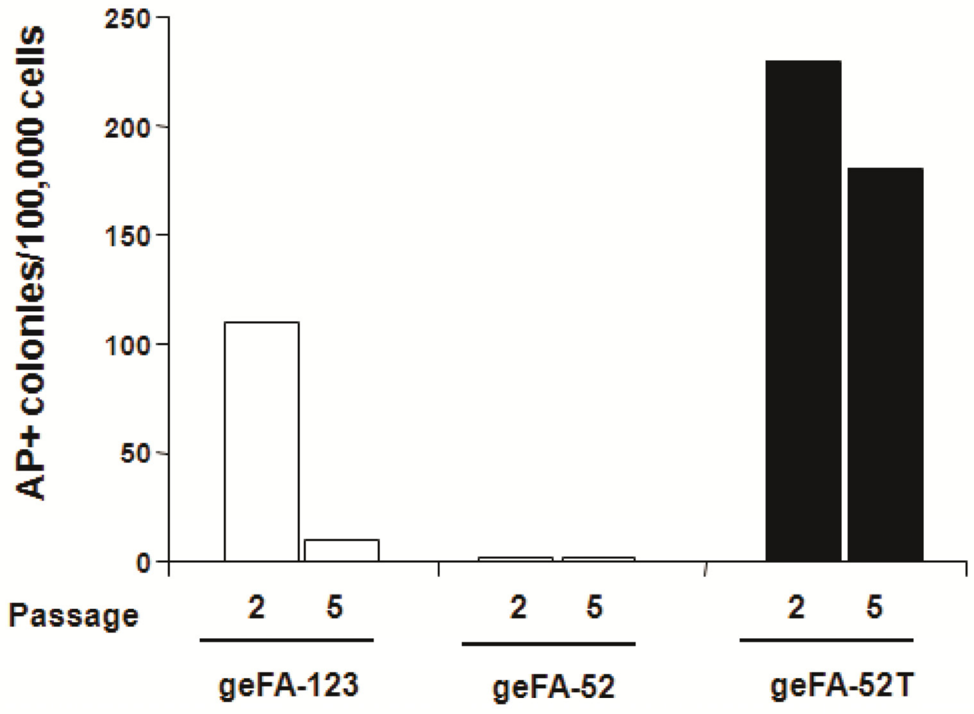

Figure S3: Reprogramming efficiency in gene edited fibroblasts from FA patients after transduction with hTERT-LV. The efficiency of reprogramming was calculated by measuring phosphatase alkaline positive (AP^+) colonies obtained from $1x10^4$ fibroblasts transduced with STEMCCA-LV. The number of alkaline phosphatase positive colonies was analyzed at two and 5 passages after transduction. White bars represent primary fibroblasts and black bars fibroblasts immortalized with telomerase.
