# Supplementary material for: Targeted gene therapy and cell reprogramming in Fanconi anemia
Source: EMBO Mol Med. 2014 May 23;6(6):835–48. doi: 10.15252/emmm.201303374 (PMC4203359; doi:10.15252/emmm.201303374)
Supplement: Supplementary file 4 — Supplementary Figure S4 [file emmm0006-0835-sd4.pdf]

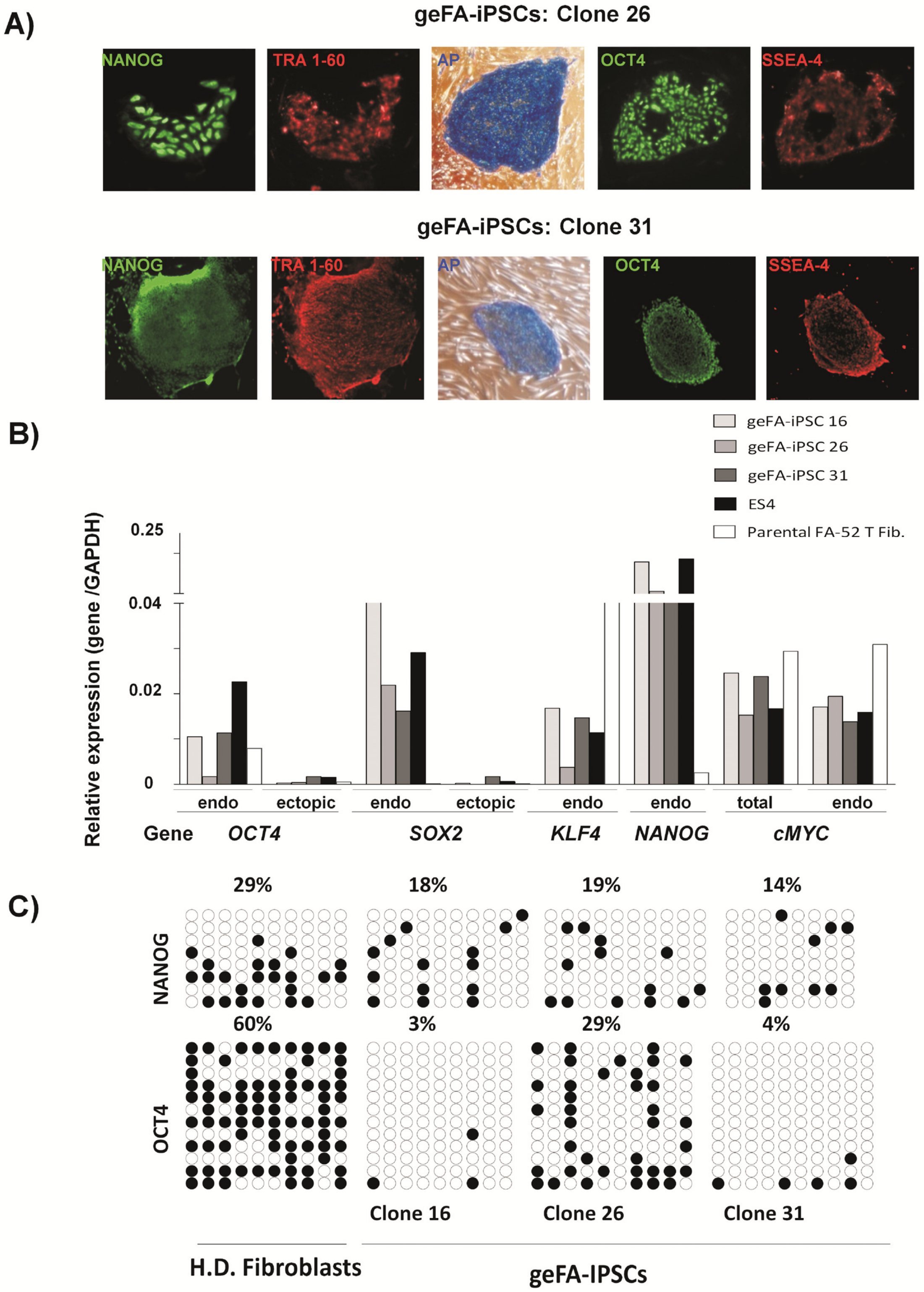
A)
geFA-iPSCs: Clone 26
NANOG
TRA 1-60
AP
OCT4
SSEA-4
geFA-iPSCs: Clone 31
NANOG
TRA 1-60
AP
OCT4
SSEA-4
B)
geFA-iPSC 16
geFA-iPSC 26
geFA-iPSC 31
ES4
Parental FA-52 T Fib.
Relative expression (gene /GAPDH)
0.25
0.04
0.02
0
endo
ectopic
endo
ectopic
endo
endo
total
endo
Gene
OCT4
SOX2
KLF4
NANOG
cMYC
C)
NANOG
29%
18%
19%
14%
OCT4
60%
3%
29%
4%
Clone 16
Clone 26
Clone 31
H.D. Fibroblasts
geFA-IPSCs

Figure S4: Pluripotency characterization of gene-edited FA-A iPSCs. A) Expression of TRA1-60, SSEA-4, OCT4 and NANOG pluripotency markers by immunofluorescence staining of gene edited iPSC clones 26 and 31. **B)** RT-qPCR analysis of *NANOG*, *OCT4*, *SOX2*, *KLF4* and *cMYC* in geFA-iPSCs (clones 16, 26 and 31). Levels of expression were normalized to a constitutive gene (GADPH). **C)** Bisulfite sequencing analysis of *OCT4* and *NANOG* promoters in ge-FA-iPSCs 16, ge-FA-iPSCs 26 and ge-FA-iPSCs 31 in comparison with healthy donor (H.D.) fibroblasts. White circles represent unmethylated CpG dinucleotides while black circles represent methylated CpG dinucleotides. Total percentage of methylated CpG of all the test clones is also shown.
