# Supplementary material for: Targeted gene therapy and cell reprogramming in Fanconi anemia
Source: EMBO Mol Med. 2014 May 23;6(6):835–48. doi: 10.15252/emmm.201303374 (PMC4203359; doi:10.15252/emmm.201303374)
Supplement: Supplementary file 5 — Supplementary Figure S5 [file emmm0006-0835-sd5.pdf]

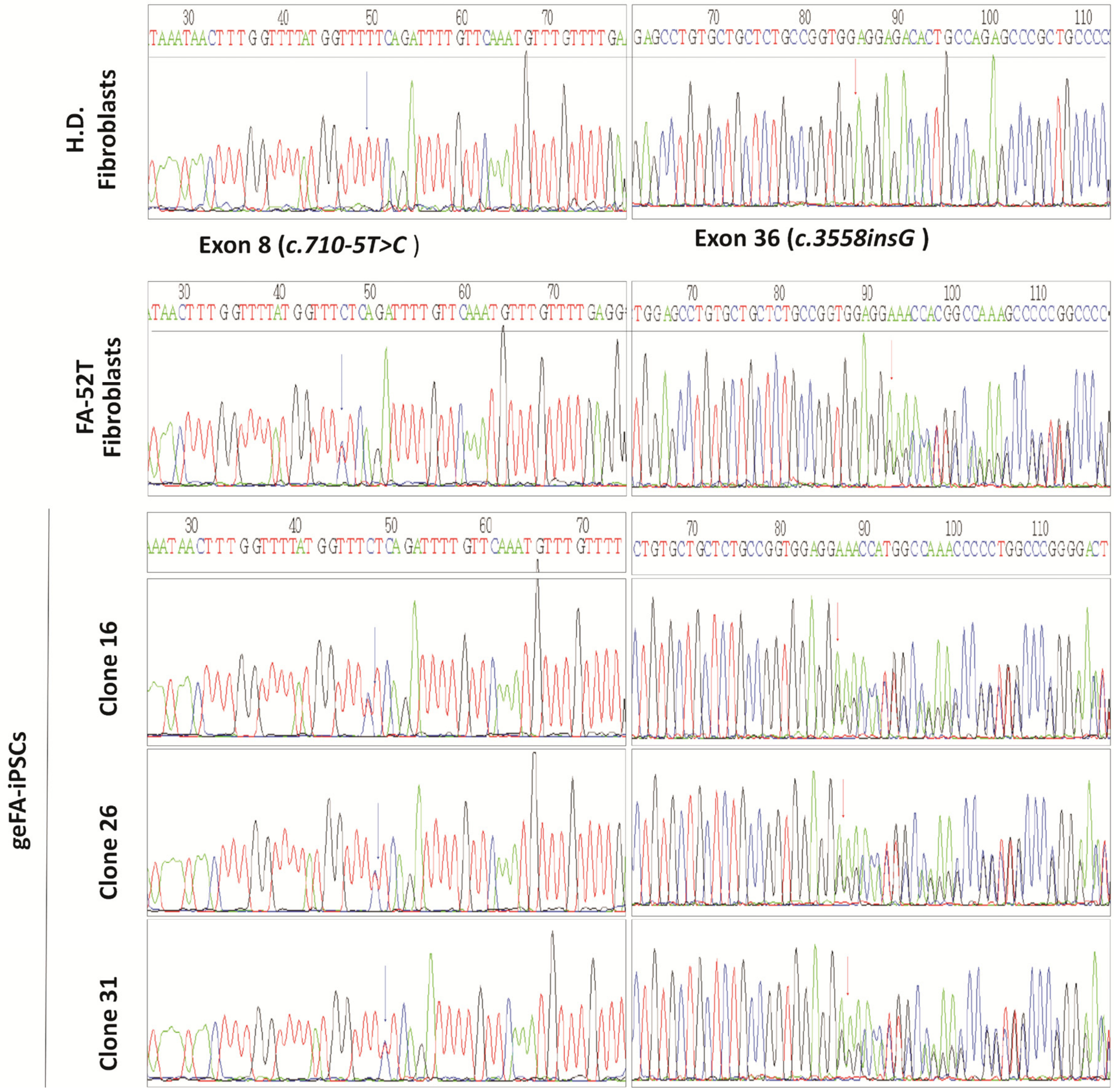

Figure S5: Molecular identity and integrity of geFA-IPSCs clones. Sequencing analysis confirmed that the two mutations found in fibroblasts from FA-52 are also present in the different clones of geFA-IPSCs. Blue arrows indicate the presence of mutation *c.710-5T>C* (exon 8) in one allele and red arrows show the mutation *c.3558insG* present in the exon 36 of the other allele. No additional mutation was detected either in an exonic region or in the adjacent intronic regions, discarding the presence of any reverse mutation in *cis.*
