# Supplementary material for: Targeted gene therapy and cell reprogramming in Fanconi anemia
Source: EMBO Mol Med. 2014 May 23;6(6):835–48. doi: 10.15252/emmm.201303374 (PMC4203359; doi:10.15252/emmm.201303374)
Supplement: Supplementary file 6 — Supplementary Figure S6 [file emmm0006-0835-sd6.pdf]

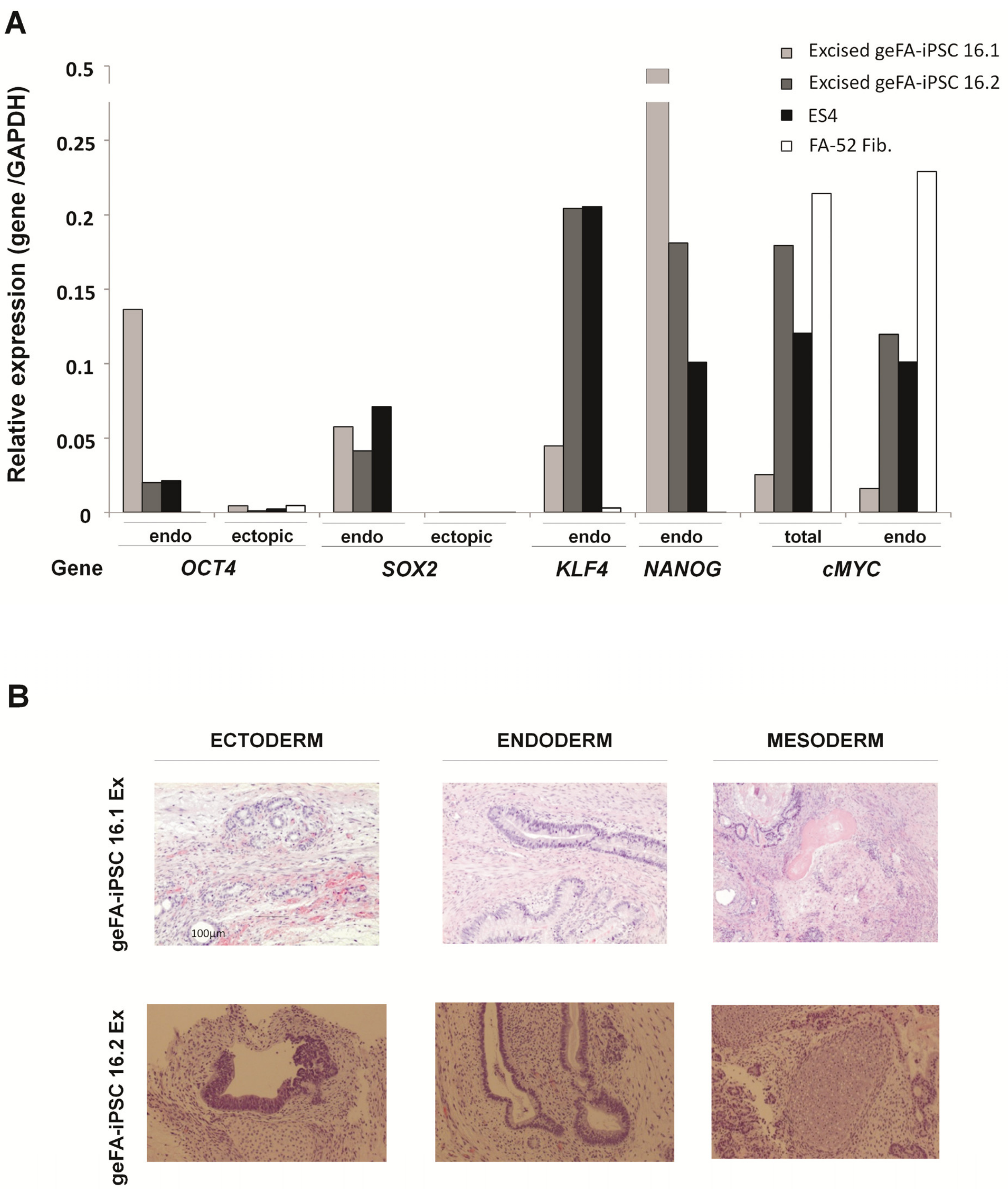

Figure S6. Characterization of two clones obtained from geFA-iPSC clone 16 after Cre-excision of the reprogramming and hTERT proviruses. A) RT-qPCR analysis of the expression of endogenous and ectopic pluripotency-related genes in excised geFA-IPSC clone 16.1 and 16.2. *NANOG*, *OCT4*, *SOX2*, *KLF4* and *cMYC.* Levels of

expression were normalized to a constitutive gene (GADPH). ES4 control line and FA-52 fibroblasts were included as controls. **B)** Hematoxylin and eosin staining of teratoma sections generated from geFA-iPSC 16 after excision of the reprogramming and hTERT proviruses, showing tissues representing the three germ layers (ecto-, meso- and endoderm) in both excised geFA-iPSC clone 16.1 and 16.2.
