# Supplementary material for: Targeted gene therapy and cell reprogramming in Fanconi anemia
Source: EMBO Mol Med. 2014 May 23;6(6):835–48. doi: 10.15252/emmm.201303374 (PMC4203359; doi:10.15252/emmm.201303374)
Supplement: Supplementary file 7 — Supplementary Figure S7 [file emmm0006-0835-sd7.pdf]

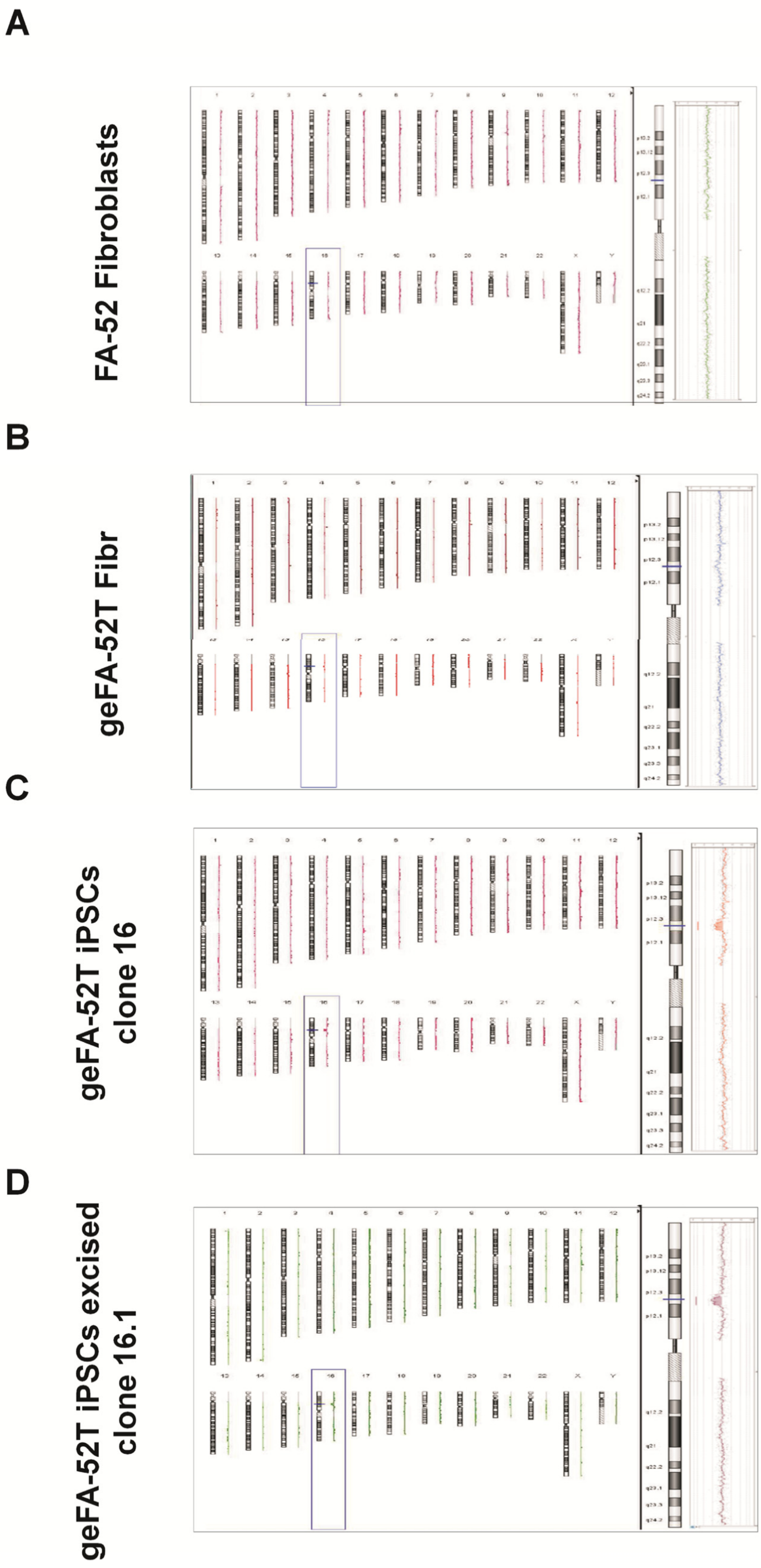
A
FA-52 Fibroblasts
B
geFA-52T Fibr
C
geFA-52T iPSCs clone 16
D
geFA-52T iPSCs excised clone 16.1

Figure S7: Comparative genome hybridization array (aCGH) analysis in fibroblasts and iPSCs from sample FA-52 after each of the *ex vivo* manipulations required for the generation of reprogramming vector-free disease-free FA iPSCs. A): aCGH analysis corresponding to expanded FA-52 fibroblasts and **B)** the same cells transduced with hTERT-LVs and subjected to gene editing. **C)** Analysis of the same sample after cell reprogramming and expansion of the corresponding iPSCs (clone 16). **D)** Analysis of the same clones after excision of the reprogramming and hTERT proviruses with Cre-recombinase.
