# Supplementary material for: Targeted gene therapy and cell reprogramming in Fanconi anemia
Source: EMBO Mol Med. 2014 May 23;6(6):835–48. doi: 10.15252/emmm.201303374 (PMC4203359; doi:10.15252/emmm.201303374)
Supplement: Supplementary file 8 — Supplementary Figure S8 [file emmm0006-0835-sd8.pdf]

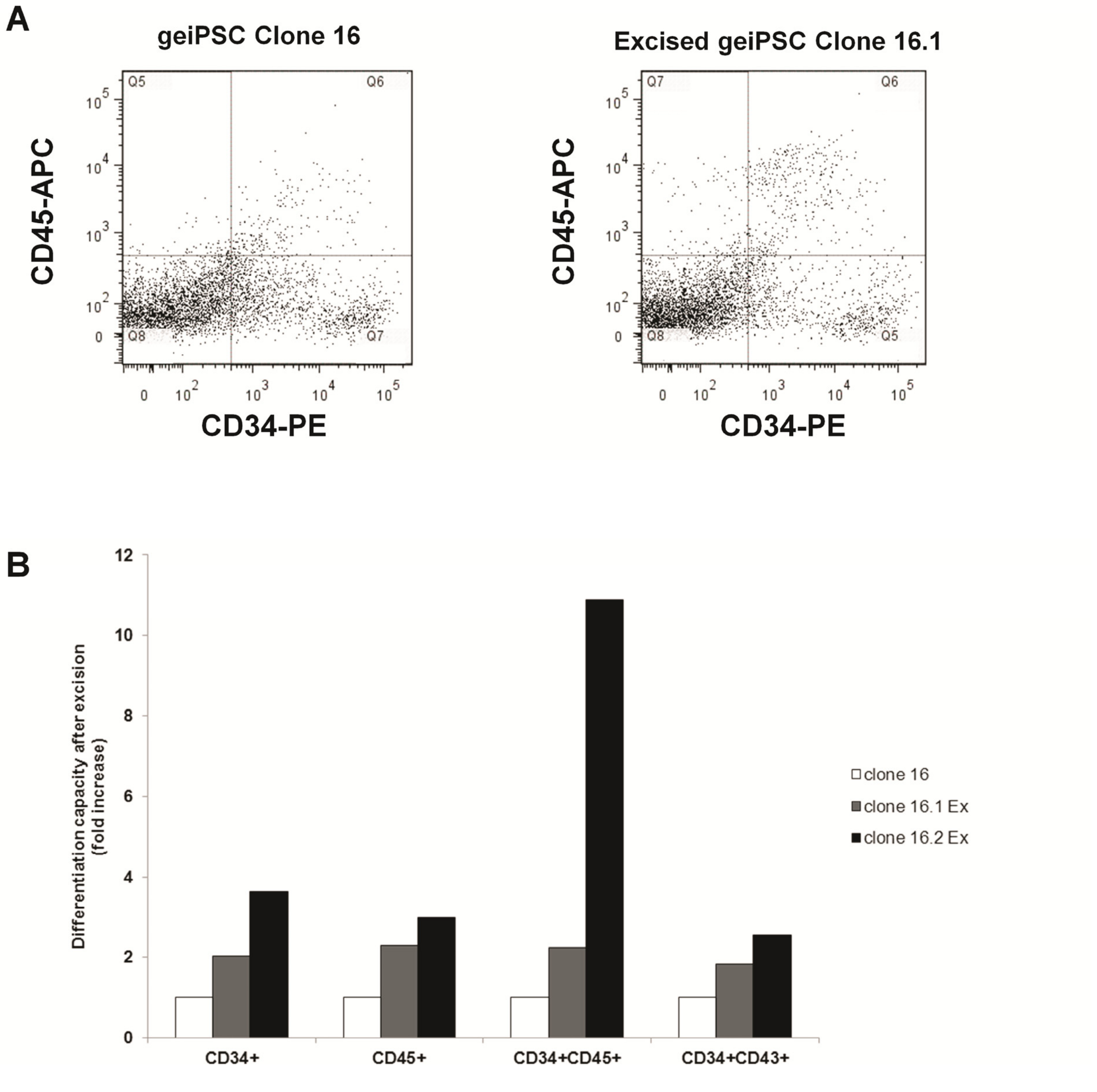


**Figure S8:** ***In vitro*** **hematopoietic differentiation of gene edited FA iPSCs. A)** Representative flow cytometry dot plots of human CD34$^+$ and CD45$^+$ hematopoietic cells obtained by *in vitro* differentiation of geFA-iPSCs 16 before and after Cre-mediated excision of OSKM factors (geFA-iPSCs Ex16.1). **B)** Improved differentiation potential in geFA-iPSCs after excision of the reprogramming cassette. The relative expression of different hematopoietic markers (CD34$^+$, CD45$^+$ CD34$^+$CD43$^+$, CD34$^+$CD45$^+$) was analyzed in geFA-IPSCs 16 and two different excised clones (geFA-iPSCs Ex16.1 and Ex 16.2).
