# Supplementary material for: Targeted gene therapy and cell reprogramming in Fanconi anemia
Source: EMBO Mol Med. 2014 May 23;6(6):835–48. doi: 10.15252/emmm.201303374 (PMC4203359; doi:10.15252/emmm.201303374)
Supplement: Supplementary file 9 — Supplementary Figure S9 [file emmm0006-0835-sd9.pdf]

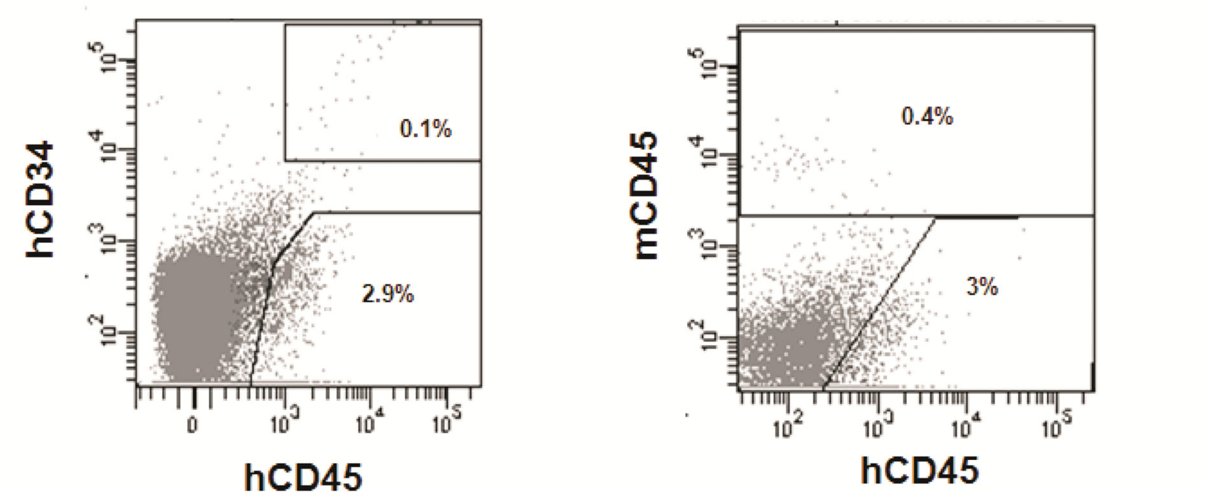

Figure S9: *In vivo* hematopoietic differentiation of gene edited FA iPSCs. The figure shows the proportion of $hCD34^+$ and $hCD45^+$ cells (Left panel) in a teratoma generated by geFA-iPSCs 16. Twelve weeks after implantation of the iPSCs with OP9 cells. To confirm the specific expression of hCD45 staining, cells were also stained with a mCD45 antibody (right panel).
