# Supplementary material for: Targeted gene therapy and cell reprogramming in Fanconi anemia
Source: EMBO Mol Med. 2014 May 23;6(6):835–48. doi: 10.15252/emmm.201303374 (PMC4203359; doi:10.15252/emmm.201303374)
Supplement: Supplementary file 10 — Supplementary Table S1 [file emmm0006-0835-sd10.pdf]

Table S1: PCR analyses of gene-edited FA-iPSC clones.

Cell Type	Clone ID	Donor IDLV (*EGFP*$^+$)	3'-*AAVS1*/Donor
geFA-iPSCs fibr	1	0.74	+
	5	0.06	- *
	11	0.38	+
	12	1.09	+
	14	0.84	+
	16	0.64	+
	17	0.41	+
	23	1.10	+
	26	1.31	+
	31	0.96	+
	33	1.77	+
	41	1.54	+
Parental Fibr.	FA-52T	0.01	-

* This clone failed to proliferate after 6 passages in culture.

Individually iPSC clones were expanded and differentiated toward fibroblastic cells during 10 days. Samples from geFA-iPSC derived fibroblasts were then analysed to evaluate the mean copy number of the donor IDLV per cell (EGFP analyses) and the specific integration of the donor cassette in the *AAVS1* locus.

geFA-iPSC fibr: Fibroblasts derived from the respective geFA-iPSC clones were expanded and used for genetic analyses.

Parental Fibr: *hTERT*-transduced parental fibroblasts from patient FA-52 were used as a control.

Donor IDLV (EGFP$^+$): Data indicates the amplification of EGFP marker gene contained in the donor IDLV. Data correspond to qPCR analyses.

***AAVS1* PCR:** The integration of the donor IDLV within the *AAVS1* locus was confirmed by PCR, according to analyses shown in figure 1F.
