# Supplementary material for: Targeted gene therapy and cell reprogramming in Fanconi anemia
Source: EMBO Mol Med. 2014 May 23;6(6):835–48. doi: 10.15252/emmm.201303374 (PMC4203359; doi:10.15252/emmm.201303374)
Supplement: Supplementary file 11 — Supplementary Table S2 [file emmm0006-0835-sd11.pdf]

Table S2: Description of the primers used in conventional PCR and qPCR.

General function	Gene	Application	Primer ID	Sequence
Gene targeting Analysis by PCR	*AAVS1*	Targeted gene addition into the AAVS1 site (5' junction)	AAVS1 F	5’-AACTCTGCCCTCTAACGCTGC-3'
			eGFP R	5’- TGGTGCAGATGAACTTCAGGG-3’
		Targeted gene addition into the AAVS1 site (3' junction)	hFANCA F	5’- AAAGCTCGTCTTTTTCTGCTGCAGT-3’
			AAVS1 R	5’-AACGGGGATGCAGGGGAACG-3’
Gene Targeting Analysis by qPCR	*eGFP*	copy number of the HR cassette	eGFP F	5’-GTAAACGGCCACAAGTTCAGC-3’
			eGFP R	5’-TGGTGCAGATGAACTTCAGGG-3’
			hAlbumin F	5’-GCTGTCATCTCTTGTGGGCTGT-3’
			hAlbumin R	5’-ACTCATGGGAGCTGCTGGTTC-3’
Copy number of the reprogramming vector Analysis by qPCR	Wpre sequence	copy number of STEMCCA vector	Wpre F	5’-GGCACTGACAATTCCGTGGT-3’
			Wpre R	5’-AGGGACGTAGCAGAAGGACG-3’
	hAlbumin gene		hAlbumin F	5’-GCTGTCATCTCTTGTGGGCTGT-3’
			hAlbumin R	5’-ACTCATGGGAGCTGCTGGTTC-3’
h*FANCA* expresión Analysis by qPCR	*hFANCA*	h*FANCA* expression	FANCA F	5’-GCTCAAGGGTCAGGGCAAC-3’
			FANCA R	5’-TGTGAGAAGCTCTTTTTCGGG-3’
			FANCA Probe	5’-FAM-CGTCTTTTTCTGCTGCAGTTAATACCTCGGT-BHQ1- 3’
	B-Actin		B-actin F	5’-ATTGGCAATGAGCGGTTCC-3’
			B-actin R	5’-CACAGGACTCCATGCCCA-3’
			B-actin Probe	5’-TexasRed-CCCTGAGGCACTCTTCCAGCCTTCC-BHQ2-3’
Expression of pluripotency genes in iPSC clones	*OCT4*	Expression of endogenous h*OCT4*	endoOCT4 F	5’-TTCGCAAGCCCTCATTTC-3’
			endoOCT4 R	5’-CCATCACCTCCACCACCT-3’

		Ectopic expression of mOct4 (from the integrated LV)	Exog mOct4 F	5'-AAGTTGGCGTGGAGACTTTG-3'
			Exog mOct4 R	5'-TACGGTAGGGAGGCGTCTT-3'
	SOX2	Expression of endogenous h*SOX2*	endoSOX2 F	5'-TGCTGCCTCTTTAAGACTAGGAC-3'
			endoSOX2 R	5'-CCTGGGGCTCAAACTTCTCT-3'
		Expression of the m*SOX2 (*from the integrated LV)	exog mSOX2 F	5-'CGCGGAGTGGAAAGAAGTA-3'
			exog mSOX2 R	5'-AAGAGGACCCGGTAGAATGC-3'
	KLF4	Expression of the endogenous h*KLF4*	endoKLF4 F	5'-GACCACCTCGCCTTACACAT-3'
			endoKLF4 R	5'-TTCTGGCAGTGTGGGTCATA-3'
	NANOG	Expression of the endogenous h*NANOG*	NANOG F	5'-TCTCCAACATCCTGAACCTCA-3'
			NANOG R	5'-TTGCTATTCTTCGGCCAGTT-3'
	cMYC	Expression of the endogenous hc*MYC*	endoMYC F	5'-GCTGCTTAGACGCTGGATTT-3'
			endoMYC R	5'-TAACGTTGAGGGGCATCG-3'
		total expression of cMyc (endongenous and from the reprogramming LV)	TotalcMYC F	5'-AGCGACTCTGAGGAGGAACA-3'
			TotalcMYC F	5'-CTCTGACCTTTTGCCAGGAG-3'
	hGADPH	Expression of hGAPDH (control gene)	hGADPH F	5'-GGCATGGACTGTGGTCATGA-3'
			hGADPH F	5' –TGCACCACCAACTGCTTAGC-3'
DNA Methylation	*OCT4 promoter*	Detect 12 CpG islands in the OCT4 promoter	OCT4 promoter F	5'-GAGGTTGGAGTAGAAGGATTGTTTTGGTTT-3'
			OCT4 promoter R	5'-CCCCCCTAACCCATCACCTCCACCACCTAA-3'
	NANOG promoter	Detect 8 CpG islands in the NANOG promoter	NANOG promoter F	5'-TGGTTAGGTTGGTTTTAAATTTTTG-3'
			NANOG promoter R	5' –AACCCACCCTTATAAATTCTCAATTA-3'
Characterization of the mutations found in patient FA-52.	*hFANCA* exon 8	Detection of the *FANCA* mutation in exon 8	FANCA exon 8 F	5'-CTGAAGTGGATGGTCTGTGCC-3'
			FANCA exon 8 R	5'-CCCGTAAATAGGTACAAACAGC-3'
	hFANCA exon 36	Detection of the *FANCA* mutation in exon 36	FANCA exon 36 F	5'-GTCATGGCTGGGGCAGCGGAG-3'
			FANCA exon 36 R	5'-TCCCTGCTCACACGAGAGG-3'
