# Supplementary material for: Targeted gene therapy and cell reprogramming in Fanconi anemia
Source: EMBO Mol Med. 2014 May 23;6(6):835–48. doi: 10.15252/emmm.201303374 (PMC4203359; doi:10.15252/emmm.201303374)
Supplement: Supplementary file 12 — Supplementary Materials and Methods [file emmm0006-0835-sd12.pdf]

SUPPLEMENTARY MATERIALS AND METHODS

Lentiviral Vector Production.

Integration-competent and Integrase-Defective Lentiviral Vectors (LVs and IDLVs, respectively) pseudotyped with the vesicular stomatitis virus G protein (VSV-G) were produced in 293T cells as previously described (Gonzalez-Murillo et al, 2010). Briefly, 293T cells were cotransfected using Fugene 6 transfection reagent (Promega) with the required transfer vector plasmid, with the pMD.Lg/RRE (for LVs) or pMD.Lg/pRRE.D64Vint (for IDLVs) packaging plasmid, pMD2.VSV-G envelope-encoding plasmid and pRSV-Rev in the following amounts: 10/5/5/5 µg DNA per 10-cm dish, respectively. Vector particles were filtered by 0.45 µm and concentrated 100-fold by ultracentrifugation. Titration was conducted either by qPCR as previously described (Charrier et al, 2010) or by HIV-1 Gag p24 immunocapture assay (Perkin Elmer) in the case of IDLVs. AdV5/35 expressing ZFNs targeting the AAVS1 locus were kindly provided by Sangamo Bioscience Inc.

Telomerase activity.

In vitro telomerase activity was measured using the TRAPEZE® RT Telomerase Detection Kit (Millipore) following manufacturer´s instructions. Protein was obtained from FA-52, FA-52T and geFA-52T fibroblasts using the CHAPs lysis buffer. Reactions were incubated at 30ºC during 30 minutes to allow the amplification of telomeric repeats by the telomerase and then subjected to a PCR amplification of telomeric products generated by qPCR. Each reaction was performed by PCR. Heat inactivated controls, a positive control and three negative controls were included: without template, without Taq polymerase and minus telomerase control.

To quantify telomerase activity a standard curve with the TSR8 template was generated following manufacturer´s instructions.

Cell reprogramming.

Gene edited FA-fibroblasts were transduced at MOI=1 with the pHAGE–STEMCCA–LoxP LV carrying the OCT4, KLF4, SOX2 and c-MYC reprogramming factors (Somers et al, 2010). Twenty four hours later, media was changed, and two days later hFGF (10 ng/ml) was added. Four days after transduction, media was replaced by hES medium

composed by DMEM-KO (Gibco), non-essential aminoacides, 1% NEEA (Lonza, Biowhitlaker), glutamax (2 mM; Gibco), 1% penicillin/streptomycin (Gibco), 10% KO-Serum Replacement (Gibco), β-Mercaptoetanol (50 µM; Gibco) and hrFGF (10 ng/ml; Prepotech). Two days later, $2x10^4$ fibroblasts were seeded in a 10 cm^2 dish onto feeder of 10^6 HFFs previously irradiated with 45 Gy. After 11 days the first colonies appeared and were individually picked to generate individual iPSC clones that were maintained as previously described (Raya et al, 2010).

Expression of pluripotency genes in iPSC clones.

Expression of lentiviral reprogramming transgenes and endogenous pluripotency-associated transcription factors were assessed by qRT-PCR. Total RNA was extracted using High pure RNA isolation kit (Roche) and reverse transcription was conducted using RETROscript kit (Life Technologies) according to the manufacturer's instructions. Real-time PCR was performed using primers for the different genes (see Table E2) and gene expression levels were normalized to hGAPDH and SYBR green reagent kits (Roche) on an Applied Biosystems® 7500 Real-Time PCR System **and analyzed with** ABIPrism 7000 software (**Applied Biosystems). Amplification conditions were** carried out at 95°C for 10 minutes, followed by 40 cycles of 95°C for 15 seconds and 60°C for 10 minutes. Relative expression of each gene compared to undifferentiated ES4 was quantified using the $2^{-\Delta\Delta Ct}$ method.

Alkaline phosphatase staining and immunofluorescence

Alkaline phosphatase staining was performed with the Vector Blue Substrate Kit (Vector Laboratories) according to the manufacturer's protocol. For immunofluorescence, cells were fixed in 4% paraformaldehyde (Sigma-Aldrich), permeabilized with 1% BSA, 10% FBS, 0.3% glycine and 0.1% Tween 20 in PBS for 1 hour at RT, and incubated with rabbit anti-NANOG (ab21624, Abcam), mouse anti-Tra-1-60 (ab16288, Abcam), rabbit anti-Oct4 (ab19857, Abcam) or mouse anti-SSEA4 (ab16287, Abcam). As secondary antibodies goat to Rabbit IgG Alexa Fluor488 (Molecular probes A11008), and goat to mouse IgG-AlexaFluor594 (Molecular probes A11005) were used. DNA was stained with 4',6-diamidino-2-phenylindole (DAPI; Roche). Immunofluorescence images were acquired with Zeiss Axioplan2 epifluorescence microscope (Carl Zeiss) equipped with an

AxioCam MRm camera (Carl Zeiss) and the photos obtained were processed with AxioVision version 4.6.3 (Carl Zeiss) and Corel Photo-Paint 11 (Corel).

Mouse studies

NOD.Cg-*Prkdc*^{scid} *Il2rg*^{tm1Wjl}/SzJ and NOD.Cg-*Prkdc*^{scid} *Il2rg*^{tm1Wjl}/SzJ mice expressing human IL-3, GM-CSF, and SF (both from Jackson laboratory) immunodeficient mice were maintained under high standard conditions (high-efficiency particulate[HEPA]-filtered air, regulated temperature of 22 °C, light/dark cycle of 12 hours, and food and ultraviolet- irradiated water *ad libitum*) and routinely screened for pathogens. All experimental procedures were carried out according to Spanish and European regulations (Spanish RD 223/88 and OM 13-10-89 of the Ministry of Agriculture, Food, and Fisheries, and European convention ETS-123 for the use and protection of vertebrate mammals used for experimentation and other scientific purposes), and approved by the animal welfare Committee of the CIEMAT (procedures HEM5/ 09 and HEM5/13).

Generation and characterization of teratomas

geFA-iPSCs cells were removed from feeder after using colagenase type IV (Gibco) and centrifuged at 200g for 5 minutes. 1-3 x 10^6 cells were resuspended in 120 µl of hES medium together with 60 µl of matrigel (BD Biosciences) and injected subcutaneously on the back of the neck of NOD.Cg-*Prkdc*^{scid} *Il2rg*^{tm1Wjl}/SzJ immunodeficient mice. After 6-8 weeks teratomas were obtained and fixed in 10% formalin (SIGMA). Paraffin sections were prepared and analyzed histologically both via hematoxilin and eosin sections and by immunofluoresence using specific antibodies to recognize tissues from all three embryonic germ layers. For immunofluorescence paraffin sections were incubated O/N at 4ºC with one of the following antibodies: monoclonal anti-actin alpha-smooth muscle-Cy3 (C6198, Sigma), rabbit anti-Brachyury (ab 20680, Abcam), rabbit anti-FOXOA2 (ab40874, Abcam), rabbit anti-neuron specific beta III tubulin (ab18207, abcam). Samples were washed and incubated with secondary antibodies goat to rabbit IgG Alexa Fluor488 (Molecular probes A11008) and goat to mouse IgG-AlexaFluor594 (Molecular probes A11005). DAPI (Roche) was used to stain DNA. Immunofluorescence images were acquired as previously explained.

Hematopietic differentiation in teratomas.

$1x10^6$ cells from geFA-IPSCs 16.2 ex clone were injected together with $5x10^5$ OP9 stromal cells into the testes of NSG-SGM3 mice (NOD.Cg-*Prkdc*^{scid} *Il2rg*^{tm1Wjl}/SzJ mice expressing human IL-3, GM-CSF, and SF; The Jackson Laboratory). 200 ng of TPO (Eurobiosciences) were also administered during two weeks via a micro-osmotic pump implanted subcutaneously for two weeks as previously described (Suzuki et al, 2013). Three months later teratomas were obtained, disgregated with collagenase IV (Sigma Aldrich) and analized by flow cytometry after staining with mouse Ly 5.1-PE, human CD34-APC and hCD45 APCCy7. Dapi was used to discard death cells.

Karyotype and chromosomal breakage analysis

Fibroblasts and geFA-iPSCs clones treated or not with Diepoxibutane (0.1µg/ml) for three days were incubated at 37ºC with 10 µg/ml colcemid during 4 hours at 37 ºC, followed by treatment with 0.56 % KCl during 15 min at 37 ºC and fixed in methanol: acetic acid (3:1). Hybridization was conducted using a protocol modified from Samper et al. (Estrada et al, 2012; Samper et al, 2002) with a Cy3-labeled LL(CCCTAA)3 PNA telomeric probe and a FITC-labeled LL(ATTCGTTGGAAACGGGA) PNA alpha satellite probe (Eurogentec). Slides were then mounted in Vectashield H-1200 mounting medium with DAPI (Vector Laboratories). 20 metaphases were scored from each sample. Identification of different chromosomal aberrations was conducted following these criteria: chromatid or chromosomal breakages: gaps in one or two chromatids whose corresponding centromere was identified, chromosome fragments: chromosome pieces (with or without telomere) whose corresponding chromosome was not easily identified, fusions: chromosomes fused by theirs ends (end-to-end fusions can be two chromosomes fused by their p arms, with or without telomeric signals [Robertsonian-like Fusion]), or two chromosomes fused by their q arms [dicentrics], the telomeric associations also were included in this category and finally, multiaberrant cells: Cells with more than one chromosomal aberration. Fluorescence images were captured with a Nikon 90i microscope fitted with a 100X 1.3 N/A planflour objective, and an Olympus monochrome digital camera (Kushima City, miyazaki, Japan). Digital images were acquired with Cytovision Genus software (Genetix, Boston, MA, USA).

Array-CGH

Genomic DNA from fibroblasts and geFA-IPSCs were hybridized using the platform KaryoNIM STEM CELL® (designed by NIMGenetics), an array-CGH of 60,000 primers distributed along the genome and focus to detect the genetic alterations related with chromosomal instability. Bioinformatic analysis was perform using the parameter ADAM-2 (window 0.5 Mb, A=6) to analyze the DNA copy number present for the different probes. It was accepted as DNA copy number variation when there were at least 5 consecutive probes. The design cover, at least with 5 probes, 395 genes and 20 regions related with oncohematologic diagnosis (http://sanger.ac.uk/genetics/CGP/Census/). The analysis resolution in the rest of the genome is about 300 kba. Parental fibroblasts from patient FA-52 were compared with a reference DNA from a healthy donor (Promega). FA-52T fibroblasts corrected by gene editing and geFA-IPSCs were compared to parental FA-52 fibroblasts. Data from array-CGH has been deposited in NCBI Gene Expression Omnibus (GEO). Accession number: GSE56790.

DNA Methylation analysis

To determine the methylation status of the CpG islands in the promoters of OCT4 and NANOG genes, genomic DNA from $1x10^6$ cells was subjected to sodium bisulfite treatment followed by bisulfite-specific PCR (BSP). *"Hot start*" PCR was performed for 35 cycles consisting on denaturation at 95°C for 1 minute, annealing at 58°C for 1 minute, and extension at 72°C for 1 minute for all primer sets. For OCT4 and NANOG promoter analysis primers previously described were used (Takahashi et al, 2007)(Table E2) to generate a product of 466 bp long with 12 CpG islands and a product of 335 bp long with 8 CpG islands, respectively. Fragments obtained by PCR were separated by electrophoresis on 2% agarose gel and cloned in a TOPO vector (TOPO-TA Cloning® *Kit for Sequencing*; Invitrogen Life Technologies, Paisley, UK) for DNA sequencing. Sequences were analyzed using the Methaprimer software.

Vector copy number

One hundred ng of genomic DNA was isolated from fibroblasts derived from geFA-iPSCs using a DNeasy blood and tissue kit (Qiagen). qPCR was used to calculate the

copy number of HDR with EGFP primers **and hAlbumin gene (hALB)**. To detect the copy number of the STEMCCA LV **primers for Wpre sequence and hALB were used (Table E2). In both cases qPCR was conducted in** an Applied Biosystems® 7500 Real-Time PCR System **(Applied Biosystems) as previously described** (Charrier et al, 2007). Standard curves for Wpre or EGFP and hALB were generated by serial dilutions of the pRRLcpptPGKGFPWPRE.Alb LV plasmid (from 10^7 to 10^2). Genomic DNA from HT4A cells contaning 1 copy of the pRRLcpptPGKGFPWPRE.Alb LV per cell was included as a reference control (Charrier et al, 2007). Genes with a Ct value ≥37 were excluded from the analyses.

Colony-forming unit assays.

EB-differentiated geFA-iPSCs populations ($1x10^5$ cells) were plated in methylcellulose H4535 (Stem Cell Technologies) plus hrEPO (8U/ml; Eprex) and incubated at 37°C and 5% CO_2 for 14 days. Colonies were scored based on morphological characteristics. Microphotographies were obtained in an inverted microscope (Olympus IX70 WH10x/22; objective 4x). Resistance to Mitomycin C (MMC) was conducted by incubating the cells with 10 nM of MMC.

Identity of geFA-IPSCs.

To confirm the identity of geFA-IPSC mutations with those found in parental fibroblasts from FA-52 (exon 8:c.710-5T>C and exon 36: c.3558insG), primers for the intronic regions flanking mutated exons in *hFANCA* gene were used (**Table E2**) (Castella et al, 2011). Conventional PCR using Taq polymerase (Invitrogen) was performed at 94ºC for 5 minutes, 35 cycles consisting on denaturation at 94°C for 20 seconds, annealing at 58°C for 30 seconds, and extension at 72°C for 40 seconds and a final cycle at 72ºC for 5 minutes for each primer pair. The products were separated by electrophoresis on 2% agarose gel, purified using NucleoSpin Extract II kit (Macherey-Nagel) and sequenced by means of ABI 3730XL (Applied Biosystems). The data analysis were performed both by local alignment using the software “EMBOSS Matcher - Pairwise Sequence Alignment” (http://www.ebi.ac.uk/Tools/psa/emboss_matcher/nucleotide.html) and the genetic database “ensembl” (http://www.ensembl.org/index.html), and by the

interpretation of the chromatograms from the sequences using the software “Chromas Lite 2.01”.

Statistical analysis.

Statistical analysis was conducted using the GraphPad Prism 5 software. In all graphs bars show mean values +-SE. One-way ANOVA test with Bonferroni’s multiple comparison post-test was used to assess statistical significance of differences between groups. When applicable p values were calculated using two-tailed Student´s *t*-test analyses.

REFERENCES

Castella M, Pujol R, Callen E, Trujillo JP, Casado JA, Gille H, Lach FP, Auerbach AD, Schindler D, Benitez J et al (2011) Origin, functional role and clinical impact of Fanconi anemia FANCA mutations. Blood 117**:** 3759-3769

Charrier S, Dupre L, Scaramuzza S, Jeanson-Leh L, Blundell MP, Danos O, Cattaneo F, Aiuti A, Eckenberg R, Thrasher AJ et al (2007) Lentiviral vectors targeting WASp expression to hematopoietic cells, efficiently transduce and correct cells from WAS patients. Gene Ther 14**:** 415-428

Charrier S, Ferrand M, Zerbato M, Precigout G, Viornery A, Bucher-Laurent S, Benkhelifa-Ziyyat S, Merten OW, Perea J, Galy A (2010) Quantification of lentiviral vector copy numbers in individual hematopoietic colony-forming cells shows vector dose-dependent effects on the frequency and level of transduction. Gene Ther 18**:** 479-487

Estrada JC, Albo C, Benguria A, Dopazo A, Lopez-Romero P, Carrera-Quintanar L, Roche E, Clemente EP, Enriquez JA, Bernad A et al (2012) Culture of human mesenchymal stem cells at low oxygen tension improves growth and genetic stability by activating glycolysis. Cell death and differentiation 19**:** 743-755

Gonzalez-Murillo A, Lozano ML, Alvarez L, Jacome A, Almarza E, Navarro S, Segovia JC, Hanenberg H, Guenechea G, Bueren JA et al (2010) Development of lentiviral vectors with optimized transcriptional activity for the gene therapy of patients with Fanconi anemia. Hum Gene Ther 21**:** 623-630

Raya A, Rodriguez-Piza I, Navarro S, Richaud-Patin Y, Guenechea G, Sanchez-Danes A, Consiglio A, Bueren J, Belmonte JC (2010) A protocol describing the genetic correction of somatic human cells and subsequent generation of iPS cells. Nat Protoc 5**:** 647-660

Samper E, Fernandez P, Eguia R, Martin-Rivera L, Bernad A, Blasco MA, Aracil M (2002) Long-term repopulating ability of telomerase-deficient murine hematopoietic stem cells. Blood 99**:** 2767-2775

Somers A, Jean JC, Sommer CA, Omari A, Ford CC, Mills JA, Ying L, Sommer AG, Jean JM, Smith BW et al (2010) Generation of transgene-free lung disease-specific human induced pluripotent stem cells using a single excisable lentiviral stem cell cassette. Stem Cells 28**:** 1728-1740

Suzuki N, Yamazaki S, Yamaguchi T, Okabe M, Masaki H, Takaki S, Otsu M, Nakauchi H (2013) Generation of engraftable hematopoietic stem cells from induced pluripotent stem cells by way of teratoma formation. Mol Ther 21**:** 1424-1431

Takahashi K, Tanabe K, Ohnuki M, Narita M, Ichisaka T, Tomoda K, Yamanaka S (2007) Induction of pluripotent stem cells from adult human fibroblasts by defined factors. Cell 131**:** 861-872
